# Characterizing glucose, illumination, and nitrogen-deprivation phenotypes of *Synechocystis* PCC6803 with Raman spectroscopy

**DOI:** 10.7717/peerj.8585

**Published:** 2020-03-30

**Authors:** Imen Tanniche, Eva Collakova, Cynthia Denbow, Ryan S. Senger

**Affiliations:** 1 Department of Biological Systems Engineering, Virginia Polytechnic Institute and State University (Virginia Tech), Blacksburg, VA, United States of America; 2 School of Plant & Environmental Sciences, Virginia Polytechnic Institute and State University (Virginia Tech), Blacksburg, VA, United States of America; 3 Department of Chemical Engineering, Virginia Polytechnic Institute and State University (Virginia Tech), Blacksburg, VA, United States of America

**Keywords:** Raman spectroscopy, Cyanobacteria, Synechocystis, Microbial phenotyping, RametrixTM, Principal component analysis, Discriminant analysis

## Abstract

**Background:**

*Synechocystis sp.* PCC6803 is a model cyanobacterium that has been studied widely and is considered for metabolic engineering applications. Here, Raman spectroscopy and Raman chemometrics (Rametrix™) were used to (i) study broad phenotypic changes in response to growth conditions, (ii) identify phenotypic changes associated with its circadian rhythm, and (iii) correlate individual Raman bands with biomolecules and verify these with more accepted analytical methods.

**Methods:**

*Synechocystis* cultures were grown under various conditions, exploring dependencies on light and/or external carbon and nitrogen sources. The Rametrix™ LITE Toolbox for MATLAB® was used to process Raman spectra and perform principal component analysis (PCA) and discriminant analysis of principal components (DAPC). The Rametrix™ PRO Toolbox was used to validate these models through leave-one-out routines that classified a Raman spectrum when growth conditions were withheld from the model. Performance was measured by classification accuracy, sensitivity, and specificity. Raman spectra were also subjected to statistical tests (ANOVA and pairwise comparisons) to identify statistically relevant changes in *Synechocystis* phenotypes. Finally, experimental methods, including widely used analytical and spectroscopic assays were used to quantify the levels of glycogen, fatty acids, amino acids, and chlorophyll a for correlations with Raman data.

**Results:**

PCA and DAPC models produced distinct clustering of Raman spectra, representing multiple *Synechocystis* phenotypes, based on (i) growth in the presence of 5 mM glucose, (ii) illumination (dark, light/dark [12 h/12 h], and continuous light at 20 µE), (iii) nitrogen deprivation (0–100% NaNO_3_ of native BG-11 medium in continuous light), and (iv) throughout a 24 h light/dark (12 h/12 h) circadian rhythm growth cycle. Rametrix™ PRO was successful in identifying glucose-induced phenotypes with 95.3% accuracy, 93.4% sensitivity, and 96.9% specificity. Prediction accuracy was above random chance values for all other studies. Circadian rhythm analysis showed a return to the initial phenotype after 24 hours for cultures grown in light/dark (12 h/12 h) cycles; this did not occur for cultures grown in the dark. Finally, correlation coefficients (*R* > 0.7) were found for glycogen, all amino acids, and chlorophyll a when comparing specific Raman bands to other experimental results.

## Introduction

Cyanobacteria constitute a widespread group of Gram-negative bacteria (Stanier & Cohen-Bazire, 1977; Waterbury, 2006), and they are the only prokaryotes capable of plant-like oxygenic photosynthesis (Heidorn et al., 2011). Among the diverse cyanobacterial strains, *Synechocystis sp.* PCC6803 is the most extensively studied model organism (Knoop et al., 2010). It has been genetically well characterized and its genome fully sequenced (Kaneko et al., 1996), annotated, and available at CyanoBase (Nakamura, Kaneko & Tabata, 2000). In addition, biochemical and physiological data are well established (Angermayr et al., 2009), and a genome-scale metabolic model has been produced (Nogales et al., 2012). The ability of *Synechocystis* to grow autotrophically (fast growth compared to plants), and the ease and high efficiency of genetic engineering make this blue–green algae an attractive candidate for biotechnological and metabolic engineering applications (Angermayr et al., 2009; Heidorn et al., 2011). The most relevant applications include sustainable production of commodity chemicals (e.g., isoprene, ethylene), biofuels (e.g., ethanol, butane, fatty acids and fatty alcohols), biomaterials, and health-related compounds (Ducat, Way & Silver, 2011; Heidorn et al., 2011; Yu et al., 2013). *Synechocystis sp.* PCC6803 has no specific nutritional demands as it has the ability to adapt different growth modes, going from fully autotrophic, in the absence of an added fixed-carbon source, to fully heterotrophic (Vermaas, 1996), and even chemoheterotrophic when exposed to short periods of blue-light (Anderson & McIntosh, 1991). The oxygenic photosynthesis is achieved mainly by two multi-subunit complexes, photosystem I and photosystem II, which are embedded in the thylakoid membrane (Yao Danny, Brune Daniel & Vermaas Wim, 2012) and allow adaptation to daily fluctuations in nutrients and light. As a result, a circadian rhythm is observed in *Synechocystis sp.* PCC6803 with an approximate period length of 24 h in response to daily environmental changes (Tu Benjamin & McKnight Steven, 2006). This oscillatory behavior has been well-studied and showed that this cyanobacterium carries out photosynthesis and glycogen synthesis in the light and respiration and glycogen degradation in the dark (Whitton, 1992; Kucho et al., 2005; Van Alphen & Hellingwerf, 2015; Saha et al., 2016).

Although acclimation of *Synechocystis* to the nutrient availability in the environment has been evaluated at the transcriptional and physiological levels (Hihara et al., 2001; Yang, Hua & Shimizu, 2002; Imamura et al., 2006; Takahashi, Uchimiya & Hihara, 2008; Beck et al., 2014; Saha et al., 2016), the approaches used were time and resource-demanding. In the effort to develop a method, by which *Synechocystis* (or any microorganism) can be rapidly and inexpensively identified/characterized, we chose to evaluate *Synechocystis* phenotypes of the circadian rhythm with Raman spectroscopy and Rametrix™ (Fisher et al., 2018). This powerful analytical technique has been applied to a wide range of biological samples (Das & Agrawal, 2011) including whole-cell bacteria (Jarvis & Goodacre, 2004; Gaus et al., 2006; Pahlow et al., 2015). Raman spectroscopy is a non-destructive method and requires minimal or no sample preparation. The biological sample is excited by a monochromatic laser and the obtained spectrum shows the intensity of the Raman scattered radiation as a function of wavenumber, and spectra are extremely sample-specific (Mahadevan-Jansen et al., 1998; Crow et al., 2003; Movasaghi, Rehman & Rehman, 2007) and represents a snapshot of molecular composition. A major challenge with Raman spectroscopy is to deconvolute the complex Raman signal to extract molecular composition information. Nonetheless, Raman has been proven a rapid and reliable method for the detection and identification of microorganisms (Nelson, Manoharan & Sperry, 1992; Pahlow et al., 2015). It has also enabled the detection of phenotypic changes of *Escherichia coli* upon exposure to different alcohols (Zu et al., 2014; Freedman et al., 2016) and antibiotics (Athamneh et al., 2014). Furthermore, it has been used in conjunction with peptide-guided Surface-Enhanced Raman Scattering (pgSERS) probes to locally characterize sub-cellular compartment composition (Athamneh & Senger, 2012).

In the present study, we used Raman spectroscopy to explore the phenotypic changes related to circadian rhythm in *Synechocystis sp.* PCC6803 under different growth conditions: (i) autotrophic (i.e., photosynthesis in light/dark cycles [12 h/12 h]), (ii) photoautotrophic (i.e., continuous light at 20 µE), (iii) mixotrophic (i.e., growth with 5 mM glucose in light/dark [12 h/12 h] cycles), (iv) photomixotrophic (i.e., growth with 5 mM glucose in continuous light at 20 µE), and (v) under nitrogen limitation (0–100% NaNO_3_ of native BG-11 medium) in continuous light (20 µE). We also included (vi) an autotrophic culture grown in the dark (i.e., dark autotrophic) and (vii) a heterotrophic culture grown on 5 mM glucose and in the dark (i.e., dark heterotrophic) as a controls. This research tested the hypothesis that Raman spectroscopy and chemometric analysis using Rametrix™ LITE (Fisher et al., 2018) and PRO (Senger & Robertson, 2020) Toolboxes for MATLAB® and statistical analyses could distinguish between different *Synechocystis* phenotypes arising from the above experimental conditions. This included determining levels of some metabolites and macromolecules (glycogen, amino acids, fatty acids and chlorophyll) from Raman spectra and validating with accepted analytical methods. *Synechocystis* was the subject of this study because it has industrial importance and is a model organism for other cyanobacteria.

## Material and Methods

### Cyanobacterial strain

Axenic kanamycin resistant mutants of *Synechocystis sp.* PCC6803 (ATCC® 27184™, a glucose-tolerant strain) were generated (Heidorn et al., 2011; Tanniche, 2019) and used in all experiments.

### Culture media and growth conditions

*Synechocystis* cultures were grown in BG-11 medium (Rippka et al., 1978) (adjusted to pH 7.0) containing different amounts of nitrogen sources and glucose, and cultures were grown under different light settings. All cultures were grown in biological triplicate at 25 °C, 50 mL volume in 125 mL flasks, agitated at 140 rpm, with 15 µg/mL kanamycin, ambient CO_2_, and with 20 µE artificial light (where applicable). Autotrophic conditions were achieved by growing cells in the minimal BG-11 medium and light to alternate light/dark (or day/night) cycles (12 h/12 h). Photoautotrophic conditions were obtained by growing cells in the minimal BG-11 media under continuous artificial light (20 µE). Mixotrophic conditions consisted of growth under light/dark cycles (12 h/12 h) in BG-11 supplemented with 5 mM glucose. Photomixotrophic conditions consisted of BG-11 medium supplemented with 5 mM glucose and growth under continuous artificial light (20 µE). Nitrogen deprivation conditions consisted of BG-11^0^ medium (BG-11 medium without nitrate) supplemented with different levels of NaNO_3_ (0.88 mM, 1.76 mM, 3.52 mM, 8.8 mM, 13.2 mM, 16.72 mM and 17.6 mM corresponding to 5%, 10%, 20%, 50%, 75%, 95%, and 100% of nitrate in the BG-11 medium), where 17.6 mM corresponds to an unaltered BG-11 medium. Cultures exposed to nitrogen deprivation were grown in continuous light (20 µE). Dark autotrophic and dark heterotrophic conditions were identical to autotrophic and mixotrophic conditions, respectively, except that the cultures were kept in the dark through the duration of the experiments. Culture growth was monitored by optical density at 730 nm (OD_730_). All cells were grown to the exponential phase (OD_730_ ≈ 1.0) and harvested by centrifugation. For the circadian rhythm phenotype dynamics experiments, *Synechocystis* cells were grown to the log phase in BG-11 media, with or without glucose. An aliquot of cells (5–10 µl) was centrifuged every 2 h for 24 h prior to analyses.

### Raman spectroscopy

Cells (5 µl of culture; three biological replicates) were air dried on aluminum foil at room temperature prior to analysis. Raman spectroscopy was performed using an Agiltron PeakSeeker PRO-785 (Agiltron, Woburn, MA) Raman microscope. The spots of dried cells were imaged using a 10× objective, and the following Raman settings were used: 785 nm (30 mW) laser excitation for 5 s with spectral resolution of 13 cm^−1^. Twenty spectra were acquired and averaged per sample by focusing on different regions of the dried spot of cells.

### Rametrix™ and statistical analyses

Raman data were collected/visualized using RSIQ™ software (Agiltron) and analyzed further with the Rametrix™ LITE (Fisher et al., 2018) and PRO (Senger & Robertson, 2020) Toolboxes for MATLAB®. All calculations were performed using MATLAB® r2018a (Mathworks; Natick, MA) and required the Statistics and Machine Learning Toolbox.

The Rametrix™ LITE Toolbox was used to process all spectra, perform principal component analysis (PCA), and discriminant analysis of principal components (DAPC). Processing Raman spectra consisted of: (i) averaging the 20 spectra replicates; (ii) truncating spectra to the Raman shift biological range (400–1,800 cm^−1^); (iii) baselining using the Goldindec algorithm (Liu et al., 2015); and (iv) vector normalization. Goldingdec algorithm parameters used were: (i) baseline polynomial order = 3; (ii) estimated peak ratio = 0.5; and (iii) smoothing window size = 5.

The Rametrix™ PRO Toolbox was used to further evaluate DAPC models generated by the LITE Toolbox. Here, leave-one-out DAPC model validations were performed where (i) one sample was held out of DAPC model construction, (ii) the classification of that sample was predicted by the model, and (iii) the model prediction was compared to the actual classification. This procedure was repeated so that every sample was held out of model construction and used for prediction accuracy. The following were used to convey results: (i) accuracy (the percentage of samples predicted correctly by the model), (ii) sensitivity (the true-positive prediction accuracy), and (iii) specificity (the true-negative prediction accuracy). The Rametrix™ PRO Toolbox was also used to determine the random chance values of accuracy, sensitivity, and specificity. These are calculated with random classification predictions and can vary widely based on the number of classification factors in a dataset and their distribution. In short, Rametrix™ PRO predictions must exceed the random chance values to show effectiveness of Raman spectroscopy and Rametrix™ to classify unknown samples correctly.

**Figure 1:**
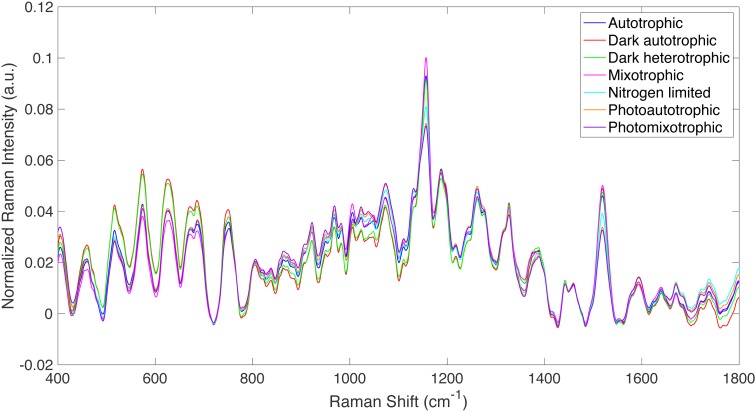
Raman spectra from all culture conditions studied. Averaged, truncated (400–1,800 cm^−1^), baselined, and vector normalized Raman spectra from all culture conditions studied.

Multi-way ANOVA and pairwise comparisons using Tukey’s honestly significant difference (HSD) method, with 99% confidence interval were used to compare Raman spectra directly. However Raman spectra consist of intensity data points over a wide range of Raman shifts (400–1,800 cm^−1^). Thus, these data-rich spectra need to be reduced from hundreds of Raman intensity values to a single value per spectrum if several spectra are to be compared by ANOVA and pairwise comparisons. This is done by calculating the multi-dimensional distance between a baselined and vector normalized Raman spectrum and a reference spectrum. In other words, the differences in Raman intensity are calculated at every Raman shift (see Fig. 1) between a Raman spectrum of *Synechocystis* under an experimental condition and a spectrum of *Synechocystis* under the control condition. In this research, the reference spectra (i.e., control condition) for each of the five sub-studies are identified in Appendix S1 in the Raw Distances and Mean Distances sheets. When comparing two spectra directly, this is referred to as the Total Spectral Distance (TSD), and it was calculated for each spectrum in this research according to Eq. (1),

(1) \begin{eqnarray*}& & TSD=\sum _{i=400}^{1,800}\sqrt{{ \left( {S}_{x,i}-{S}_{reference,i} \right) }^{2}}\end{eqnarray*}

where *S*_*x*,*i*_ is the Raman intensity value of spectrum *x* at Raman shift *i* (where *i* goes from 400–1,800 cm^−1^). The term *S*_*reference*,*i*_ represents the Raman intensity value of the reference (or control) spectrum over the Raman shift range. The calculation was repeated for all *x* spectra being analyzed. The distance calculation was also applied to principal components (PCs) from PCA analysis by the Rametrix™ LITE Toolbox according to Eq. (2), where the first five PCs (usually representing over 95% of the dataset variations) were used. This is referred to as the Total Principal Component Distance (TPD). Here, *P*_*x*,*i*_ is the value of the *i*th principal component of spectrum *x*. A more detailed description of the TPD calculation and its use in ANOVA and pairwise comparisons has been published (Senger et al., 2019).

(2) \begin{eqnarray*}& & TPD=\sum _{i=1}^{5}\sqrt{{ \left( {P}_{x,i}-{P}_{reference,i} \right) }^{2}}\end{eqnarray*}

Finally, this formula was applied to canonical values (*C*_*x*,*i*_) resulting from DAPC models built for *x* spectra using the Rametrix™ LITE Toolbox. This is referred to as the Total Canonical Distance (TCD) and is given in Eq. (3). The TCD was calculated from the top five canonicals in the DAPC model.

(3) \begin{eqnarray*}& & TCD=\sum _{i=1}^{5}\sqrt{{ \left( {C}_{x,i}-{C}_{reference,i} \right) }^{2}}\end{eqnarray*}

### Estimation of total glucose (glycogen and free glucose) levels

Glycogen levels were determined with a previously published protocol (Osanai et al., 2011). Briefly, lyophilized cells (0.5 mg) were suspended in 100 µl of 3.5% (v/v) sulfuric acid solution and boiled for 40 min. Glucose produced by acid hydrolysis was assayed spectrophotometrically at 635 nm using 6% o-toluidine solution.

### Amino acid and fatty acid analyses

Amino acids and fatty acids were analyzed as AccQ-Tag™ derivatives and fatty acid methyl esters (FAME) using Waters ultra-performance liquid chromatography (UPLC) and gas chromatography coupled with flame ionization detection (GC-FID), respectively, as described (Collakova et al., 2013). Briefly, 1 mg of lyophilized *Synechocystis* cells was subjected to biphasic extractions using 10 µg heptadecanoic acid (C17:0) and 50 mM norvaline as internal standards, followed by protein and lipid hydrolysis and derivatizations prior to UPLC and GC-MS analyses. Amino acids were analyzed on a Waters Acquity™ H-class UPLC system (Waters Corporation, Milford, MA) using fluorescent detection, while FAME on an Agilent 7890A series GC and 5975C series single quadrupole MS (Agilent Technologies, Santa Clara, CA).

### Chlorophyll a measurement

Lyophilized cells were suspended in 100% methanol and disrupted for 5 min with a bead beater. Chlorophyll a was determined spectrophotometrically at 665 nm and 720 nm after 20 min of incubation in the dark at 4 °C (Sinetova et al., 2012).

### Public availability

All Raman spectra (as raw *.SPC spectra files) and raw data measurements from analytical experiments are included as a Supplemental Data set. The Rametrix™ LITE Toolbox is available through GitHub (https://github.com/SengerLab/RametrixLITEToolbox). The Rametrix™ PRO Toolbox is also shared through GitHub (https://github.com/SengerLab/RametrixPROToolbox). Both are shared under license agreement.

## Results

### Raman spectroscopy of Synechocystis grown under different conditions

Raman spectroscopy was used to detect specific phenotypic changes in *Synechocystis sp.* PCC6803 under the following growth conditions: (i) autotrophic, (ii) mixotrophic, (iii) photoautotrophic, (iv) photomixotrophic, (v) nitrogen deprivation, (vi) dark autotrophic (control), and (vii) dark heterotrophic (control). Cells were analyzed by Raman microscopy, and the resulting spectra were processed using the Rametrix™ LITE (Fisher et al., 2018) and PRO (Senger & Robertson, 2020) Toolboxes in MATLAB®. Averaged, truncated (400–1,800 cm^−1^), baselined, and vector normalized Raman spectra are shown in Fig. 1 for the different growth conditions defined above. While the overall spectral signatures remained consistent with each other, Raman spectroscopy was able to detect significant differences in cell phenotypes that were induced by the different growth conditions. These differences were analyzed further using PCA and DAPC in the Rametrix™ LITE Toolbox, and the Rametrix™ PRO Toolbox was used to determine if these phenotypes (i.e., growth conditions) were predictable from a Raman spectrum alone (when no other information is given). Furthermore, the phenotype changes were further validated through more traditional measurements (e.g., UPLC, GC-FID, spectroscopy), as described in the Materials and Methods section. Thus, the remainder of the Results section details two different types of analyses: (i) chemometric phenotype analyses by Rametrix™ and (ii) correlation of individual Raman bands with metabolites and their levels analyzed by well-established analytical approaches.

### Chemometric phenotype analyses by Rametrix™

#### Glucose-induced phenotypes

**Figure 2:**
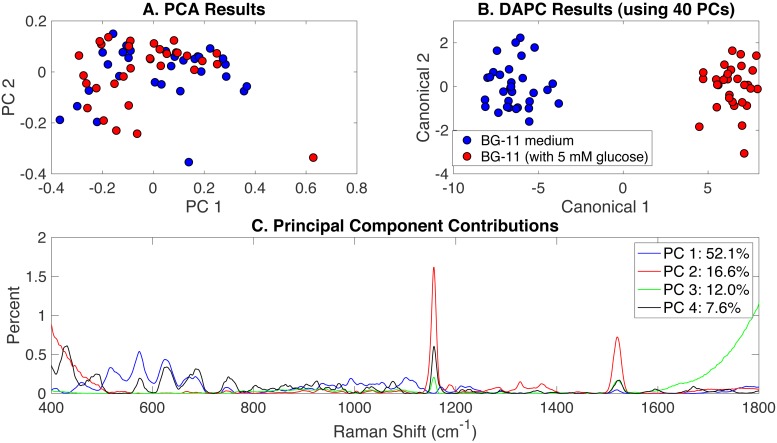
Rametrix™ models for the glucose-induced phenotypes study. (A) PCA results, (B) DAPC results when using 40 PCs, and (C) Raman shift contributions to the differences between the groups in PCA.

To investigate the effect of glucose addition to BG-11 medium on *Synechocystis* phenotypes, Rametrix™ was used to analyze the autotrophic, mixotrophic, photoautotrophic, and photomixotrophic growth. These different growth modes were assigned BG-11 medium and BG-11 (with 5 mM glucose) classifications. PCA, DAPC, and principal component contribution results generated by the Rametrix™ LITE Toolbox are given in Fig. 2. PCA of Raman spectra (Fig. 2A) demonstrated no apparent clustering in the first two PCs, which comprised over 68% of the dataset variance. Application of DAPC (Fig. 2B), however, revealed two marked clusters, based on the presence of glucose in the culture medium, when using 40 PCs (representing over 99.9% of the dataset variance). These two clusters suggest cellular phenotype differences could exist as a result of adding glucose to BG-11 medium. Next, the contributions of different Raman shifts to the PCA and DAPC results were examined. This analysis lends to discovering molecular differences between clusters of Raman spectra. In this case, it provided information about the molecular differences between cultures grown in the presence and absence of glucose. The Raman shift contributions between the two groups in PCA are shown in Fig. 2C, and the contributions leading to the separations in DAPC are shown in Appendix S1, Fig. S1. Raman band assignments were made based on a popular published library (Movasaghi, Rehman & Rehman, 2007). Specific lists of Raman band contributions and molecular assignments (for both PCA and DAPC) are given in Appendix S1, and highlights are given in Table 1.

**Table 1:** Raman shift contributions to PCA and DAPC models for selected studies.

**Study**	**Model**	Biomolecules and Raman bands (cm^−1^)
Glucose-induced	PCA	Sterols and phosphatidylinositol (430 and 575 cm^−1^, respectively); protein (750, 1,102, and 1,156 cm^−1^); and carotenoids (1,156, 1,520 cm^−1^)
Glucose-induced	DAPC	Sterols and phosphatidylinositol (430, 704, 778 cm^−1^); protein and amino acids (509, 632, 1,052, 1,245, 1,403 cm^−1^); DNA/RNA (664, 747 cm^−1^), and lipids (1,305 cm^−1^)
Illumination-induced	PCA	Sterols and phosphatidylinositol (430, 516, 574 cm^−1^) and protein/carotenoids (1,156, 1,520 cm^−1^)
Illumination-induced	DAPC	Phosphatidylinositol (509, 776 cm^−1^), phospholipids (719, 1,328 cm^−1^), lipids (1,063, 1,074 cm^−1^), glycogen (1,049, 1,155 cm^−1^), carotenoids (1,155 cm^−1^), porphyrin (1,620 cm^−1^), and several protein/polypeptide/amide bands (1,005, 1,155, 1,628, 1,633, 1,638 cm^−1^)
Nitrogen limitation-induced	PCA, DAPC	Protein-related bands (571, 623, 639, 1,156, 1,359 cm^−1^), carotenoids and porphyrin (1,156, 1,518, 1,520 cm^−1^)

Next, from spectral, PCA, and DAPC data, the TSD, TPD, and TCD distance values (Eqs. (1), (2) and (3)) were calculated and used in ANOVA and pairwise comparison tests. As observed here, and shown previously (Senger et al., 2019), statistical calculations with TSD and TPD are very similar, so only TPD and TCD are reported here. All distance data (both raw and mean values) are given in Appendix S1. With TPD data, ANOVA returned an insignificant *p*-value (*p* = 0.13) for the overall change in phenotypes between cultures grown in BG-11 medium and cultures grown in BG-11 with 5 mM glucose. TCD calculations (when using 40 PCs in DAPC), however, did find significant differences (*p* < 0.001), and these findings are consistent with the clustering shown in Fig. 2AB. With only 2 possible classifications (i.e., BG-11 with and without glucose), pairwise comparisons were identical to ANOVA results here.

Finally, Rametrix™ PRO was used to apply leave-one-out analysis to the DAPC clustering results in Fig. 2B. Essentially, this analysis determines the ability of the DAPC model to correctly predict the classification (i.e., BG-11 medium with or without glucose) when presented with an unknown Raman spectrum of *Synechocystis* cells. All Rametrix™ PRO results, for DAPC models constructed with different numbers of PCs, are given in Appendix S1, and the best-performer DAPC model is summarized in Table 2. Given that nearly equal numbers of positive and negative spectra were used, the random chance accuracy, sensitivity, and specificity for this case are all 50%. Thus, model performance far exceeded random chance. DAPC models with fewer PCs (i.e., including less dataset variability) performed worse, as did models including more than 40 PCs. Models with more than 40 PCs likely suffered from over-fitting.

**Table 2:** Summary of Rametrix™ PRO results for best-performing DAPC models.

**Study**	Numberof PCs^*^	**ClassificationPredicted**	**Accuracy**	**Sensitivity**	**Specificity**
Glucose- induced	Random chance^**^		50%	50%	50%
Glucose- induced	40 (99.9%)	BG-11 with 5 mM Glucose	95%	93%	97%
Illumination- induced	Random chance^**^		56%^***^	33%	67%
Illumination- induced	9 (99%)	Dark	89.5%	69%	90%%
Illumination- induced	9 (99%)	Light/Dark	78%	81%	75%
Illumination- induced	9 (99%)	Continuous Light	100%	100%	100%
Nitrate limitation- induced	Random chance^**^		76%	14%	86%
Nitrate limitation- induced	3 (83%)	5% (0.88 mM)	76%	67%	78%
Nitrate limitation- induced	3 (83%)	10% (1.76 mM)	95%	67%	100%
Nitrate limitation- induced	3 (83%)	20% (3.52 mM)	62%	0%	72%
Nitrate limitation- induced	3 (83%)	50% (8.8 mM)	81%	33%	89%
Nitrate limitation- induced	3 (83%)	75% (13.2 mM)	95%	67%	100%
Nitrate limitation- induced	3 (83%)	95% (16.72 mM)	52%	33%	80%
All phenotypes	Random chance^**^		76%^***^	14%	86%
All phenotypes	8 (98%)	Autotrophic	79%	25%	91%
All phenotypes	8 (98%)	Autotrophic control	86%	38%	94%
All phenotypes	8 (98%)	Dark heterotrophic	85%	38%	93%
All phenotypes	8 (98%)	Mixotrophic	80%	50%	87%
All phenotypes	8 (98%)	Nitrate limited	80%	39%	91%
All phenotypes	8 (98%)	Photoautotrophic	88%	50%	91%
All phenotypes	8 (98%)	Photomixotrophic	75%	67%	76%

**Notes.**

* Number of PCs used to build the DAPC model. The percent variance of the dataset represented is given in parentheses.

** The random chance sensitivity and specificity values were calculated statistically, not by Rametrix TM PRO. They were the same for all studies here.

*** These values are classification-dependent. The average value is given here, and individual values are given in Appendix S1.

#### Illumination phenotypes

**Figure 3:**
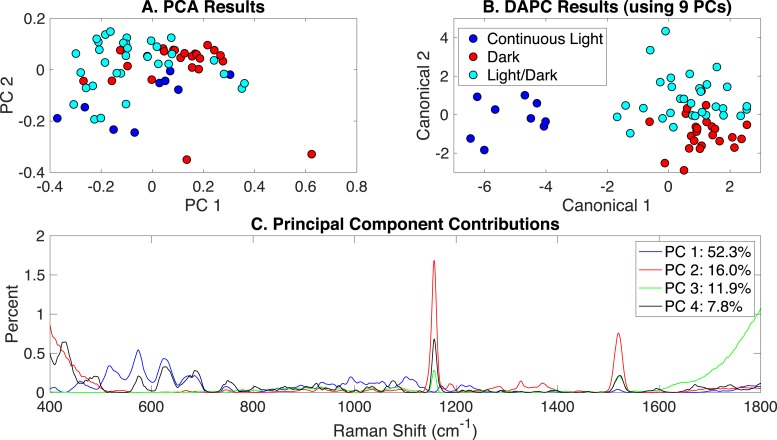
Rametrix™ models for the illumination-induced phenotypes study. (A) PCA results, (B) DAPC results when using 40 PCs, and (C) Raman shift contributions to the differences between the groups in PCA.

Similar to the previous section, Raman spectra of cultures grown in autotrophic, mixotrophic, photoautotrophic, photomixotrophic, dark autotrophic, and dark heterotrophic conditions were analyzed according to the different light settings: (i) continuous artificial light (20 µE), (ii) dark/light cycle (12h/12h), and (iii) dark. PCA, DAPC, and PC contributions generated by the Rametrix^TM^ LITE Toolbox are shown in Fig. 3. Some clustering was apparent in PCA, and this was improved significantly for the DAPC model constructed with 9 PCs, representing approximately 99% of the dataset variance. The full lists of Raman shift contributions for PCA and DAPC are given in Appendix S1, and these results for the DAPC model are shown in Fig. S2. A summary is given in Table 1. For Raman shift contributions to PCA, similar results were observed as reported for the glucose-induced phenotypes.

TSD, TPD, and TCD distances (Eqs. (1), (2) and (3)) were calculated based on spectral, PCA, and DAPC data and are given in Appendix S1 (both raw and mean values). An ANOVA test based on the type of illumination (options listed above) revealed statistically significant differences in TPD data (*p* < 0.01) and TCD data (*p* < 0.001). Pairwise comparisons showed insignificant differences in TPD values between light/dark and dark growth conditions (*p* = 0.81), somewhat significant differences between light/dark and continuous light (*p* = 0.022), and significant differences between dark and continuous light (*p* < 0.01). With TCD data (when using 9 PCs in DAPC), both pairwise comparisons involving continuous light were statistically significant (*p* < 0.001). However, the pairwise comparison for dark and light/dark was not statistically significant (*p* = 0.55). These results agree with the clustering results in Fig. 3AB, suggesting that phenotypes resulting from growth in continuous light are more easily distinguished (and separated by clustering) than those arising from growth in the dark or light/dark.

Rametrix™ PRO was used to apply leave-one-out analysis to the DAPC results in Fig. 3B. Here, Rametrix™ PRO had three classification options, dark, light/dark, or continuous light. The following example shows how the positive and negative conditions were assigned when using more than two classifications. To test the condition dark, this was assigned as the positive condition. Both light/dark and continuous light comprised the negative condition. All three conditions were tested as the positive condition in this analysis. Here, the prediction accuracy metric remains the same, the ability to classify correctly the positive and negative conditions. The sensitivity (true positive rate) holds more importance, where the specificity (true negative rate) must also be high (i.e., above random chance). With our dataset, the random chance sensitivity is 33% for each classification, and the random chance specificity is 67%. Thus, model performance sensitivity and specificity must be higher than these values if the model is truly able to classify *Synechocystis* phenotypes based on Raman spectra.

Using a DAPC model built with 9 PCs (99% of dataset variability), all conditions far exceeded the random chance sensitivity and specificity values, as shown in Table 2. In fact, the phenotypes resulting from continuous light were highly distinguishable, resulting in 100% accuracy, sensitivity, and specificity. Results for other DAPC models, constructed with different numbers of PCs, are given in Appendix S1.

#### Nitrogen limitation phenotypes

Nitrate is the most common nitrogen source used by *Synechocystis* (Flores & Herrero, 2005; Lopo et al., 2012), and we hypothesized that growth with different nitrate levels would result in significant phenotype changes observable by Rametrix™. Thus, *Synechocystis* cultures were grown in BG-11 medium with different nitrate levels (0–100% NaNO_3_ of native BG-11) and continuous light. Raman spectra were obtained of cultures at each nitrate level and were truncated (400–1,800 cm^−1^), baseline corrected, vector normalized, and averaged. These results are shown in Fig. S5 in Appendix S2. Clear differences were observed in Raman signal intensity over certain regions of the Raman shift, suggesting that there are specific changes in culture phenotypes related to nitrate deprivation.

**Figure 4:**
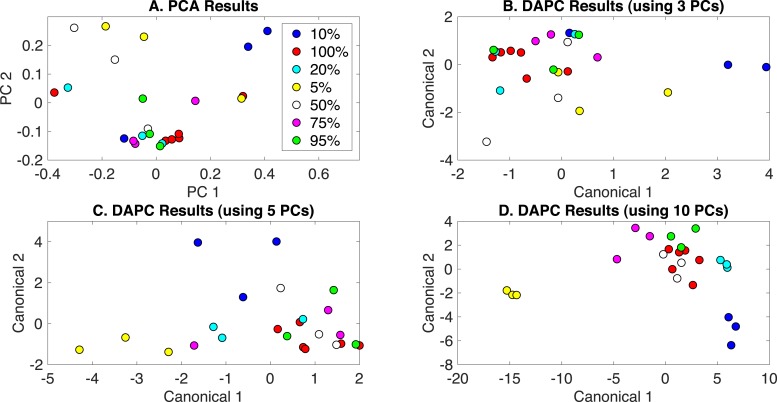
Rametrix™ models for the nitrate limitation phenotypes study. (A) PCA results, (B) DAPC results when using three PCs, (C) DAPC results when using 5 PCs, and (D) DAPC results when using 10 PCs. Percentages of nitrate in BG-11 medium are given (with 100% being native BG-11). The following are conversions between percentages and mM: 5% =0.88 mM; 10% =1.76 mM; 20% =3.52 mM; 50% =8.8 mM; 75% =13.2 mM; 95% =16.72 mM; 100% =17.6 mM.

PCA and DAPC model results are presented in Fig. 4. Again, little clustering was observed with PCA (Fig. 4A). DAPC models were built with several numbers of PCs, and analyses of these models are given in Appendix S1. DAPC models built with 3, 5, and 10 PCs (representing 83.2%, 95.6%, and 99.4% of dataset variance, respectively) are shown in Figs. 4B–4D. As more PCs were included in the DAPC model, the separation of the more nitrate deprived cultures (5% and 10%) and cultures receiving 50–100% of BG-11 levels of nitrate, which clustered together, was evident, suggesting significantly altered phenotypes may only occur at low nitrate levels. PCA and canonical contributions are given in Appendix S1 and are summarized in Table 1.

TSD, TPD, and TCD data were generated (Eqs. (1), (2) and (3)) and are available in Appendix S1. ANOVA and pairwise comparison tests were applied, and nitrate concentrations were found to be statistically significant in ANOVA (*p* < 0.001) for all TSD, TPD, and TCD data. Some pairwise comparisons showed statistical significance when considering TCD data (for DAPC model built with 3 PCs). In particular, no statistical differences were found between 100%, 95%, and 75% of nitrate present in the medium. Comparing 100% and 50% gave a *p*-value of 0.02, and comparisons with 10% and 5% gave *p*-values ≤ 0.01. Unique to this analysis, regression analysis was performed to determine the correlation between the percentage of nitrate included and calculated distances (TSD, TPD, and TCD). These results are also given in Appendix S1, and are summarized here. In particular, a correlation coefficient (R) of 0.87 was found between TSD values and the percentage of nitrate in BG-11 medium (0–100%). With TPD, a value of *R* = 0.84 was calculated. The calculation was repeated for TCD data from DAPC models built with different numbers of PCs. For a model built with 3 PCs, *R* = 0.81. For 5 PCs, *R* = 0.88, but for 10 PCs, *R* = 0.59. This further shows the incidence of overfitting when too many PCs are used to build a DAPC model.

Finally, leave-one-out analysis was applied using Rametrix™ PRO. For the nitrate limitation dataset, sensitivity must exceed the random chance sensitivity and specificity values of 14% and 86%, respectively, to demonstrate some prediction success. The best-performer DAPC model results are given in Table 2, and results for all models tested are in Appendix S1. The DAPC model built with 3 PCs (Table 2) generated an overall sensitivity of 44.4% and specificity of 86.4% across all nitrate levels, exceeding the random chance values. It is noted that some individual sensitivity/specificity values for this study (Table 2) fell below the random chance values. This would likely be remedied by expanding the number of measurements in the dataset. Models built with more PCs showed greater overall sensitivity but had inadequate specificity. For example, 5 PCs yielded sensitivity of 77.8%, but specificity of 56.5%, and 10 PCs yielded specificity of 77.8%, but specificity of 22.2% (Appendix S1).

#### Classification of all phenotypes

**Figure 5:**
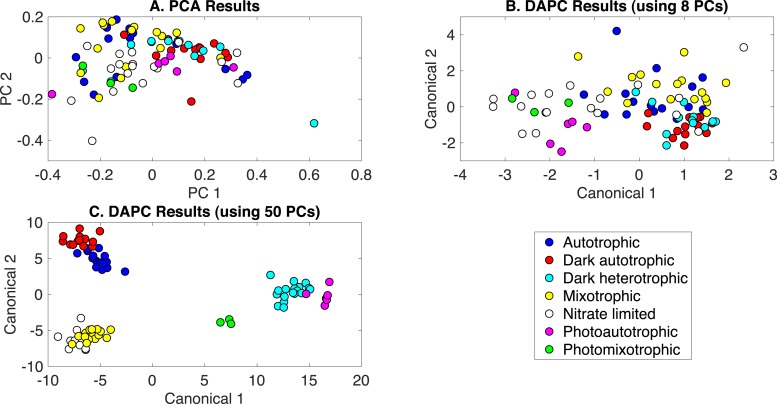
Rametrix™ models for the all phenotypes study. (A) PCA, (B) DAPC model built with eight PCs, (C) DAPC model built with 50 PCs.

The production of PCA and DAPC models with Rametrix™ LITE and validation with leave-one-out analysis with Rametrix™ PRO was repeated for a dataset consisting of all Raman spectra used in this study. This included the growth conditions: (i) autotrophic, (ii) mixotrophic, (iii) photoautotrophic, (iv) photomixotrophic, (v) nitrate limited, (vi) dark autotrophic, and (vii) dark heterotrophic. The autotrophic and mixotrophic conditions used light/dark (12 h/12 h) illumination, and the photoautotrophic, photoheterotrophic, and nitrate-limiting conditions used continuous light (20 µE). The dark heterotrophic and dark autotrophic (control) cultures were grown in the dark. PCA results are shown in Fig. 5A and, again, reveal little clustering. DAPC models were built using several numbers of PCs (the complete list is shown in Appendix S1). Models constructed with 8 and 50 PCs are shown in Figs. 5B and 5C, respectively. The clustering in Fig. 5C largely reveals clustering of phenotypes according to the presence of glucose and illumination. The following clusters were observed: (i) autotrophic and dark autotrophic conditions (no glucose; light/dark and dark), (ii) nitrate limited and photoautotrophic (no glucose; continuous light), and (iii) mixotrophic and dark heterotrophic (5 mM glucose; light/dark and dark). The only condition to not cluster was photomixotrophic, which appeared closer to the continuous light cluster than the glucose cluster in Fig. 5C.

When considering all phenotypes (listed above), statistically significant ANOVA test results (*p* < 0.001) were obtained from both TPD and TCD data. All TSD, TPD, and TCD raw and mean distance values are given in Appendix S1, along with ANOVA and pairwise comparison test results. For TPD data, pairwise comparisons showed statistical significance (*p* ≤ 0.01) between the pairs: autotrophic/mixotrophic, autotrophic/photoautotrophic, and autotrophic/photomixotrophic. For TCD data, all comparisons were statistically significant (*p* < 0.001), meaning differences were found in the cell phenotypes.

Rametrix™ PRO was used to determine if these phenotypes could be predicted from Raman spectra. With seven different classifications, the random sensitivity was calculated at 14% for each, and random specificity was calculated at 86% for each. The DAPC model built with 8 PCs (98% of dataset variance) performed the best. Results were better than random chance (except for one specificity value) and included sensitivity and specificity values (respectively) of: (i) 25% and 91% for autotrophic, (ii) 38% and 94% for autotrophic control, (iii) 38% and 93% for dark heterotrophic, (iv) 50% and 87% for mixotrophic, (v) 39% and 91% for nitrate limited, (vi) 50% and 91% for photoautotrophic, and (vii) 67% and 76% for photomixotrophic. Ultimately, prediction metrics would likely be improved with more data points given seven different possible classifications.

#### Circadian rhythm growth phenotypes

**Figure 6:**
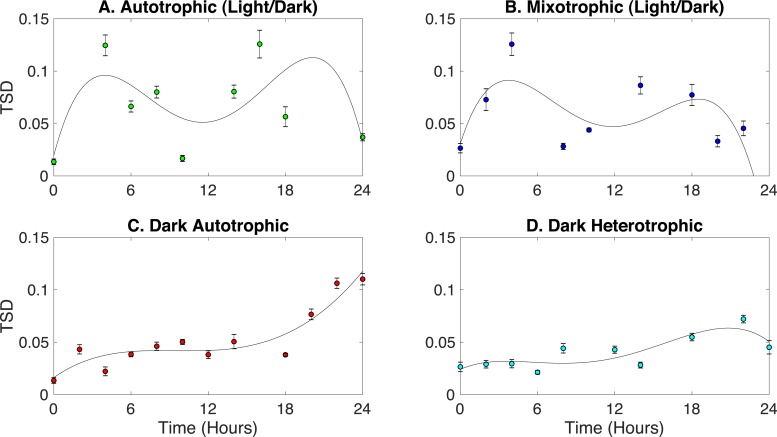
Identification of circadian rhythm phenotype changes based on TSD data for cultures grown in the following conditions: (A) autotrophic (light/dark), (B) mixotrophic (light/dark), (C) dark autotrophic, and (D) dark heterotrophic.

*Synechocystis* have a circadian rhythm to cope with daily fluctuations in available nutrients and light intensities (Tu Benjamin & McKnight Steven, 2006). Studies have shown that photosynthesis and glycogen synthesis are carried-out in the light, and respiration and glycogen degradation are adopted in the dark (Whitton, 1992; Kucho et al., 2005; Van Alphen & Hellingwerf, 2015; Saha et al., 2016). Here, Rametrix™ was employed to detect phenotypic changes related to the circadian rhythm in *Synechocystis sp.* PCC6803 grown under four different conditions: (i) autotrophic (with light/dark [12 h/12 h] cycles), (ii) mixotrophic (with light/dark [12 h/12 h] cycles), (iii) autotrophic in the dark, and (iv) heterotrophic in the dark. It was hypothesized that a circadian rhythm would be observed in cultures grown under light/dark cycles and absent from cultures grown in the dark only. For a circadian rhythm, phenotypes should deviate from its initial point during the course of the 24-hour light/dark cycle and eventually return to the starting point at the end of the cycle. Rametrix™ is well-suited to probe this cycle, and the TSD was chosen to represent phenotype changes. The TPD calculations would also work here. As a phenotype deviates from its initial phenotype, the TSD should become larger in value, and as a culture returns to its initial phenotype, the TSD value will become smaller (or very close to zero). Results for the four culturing conditions are shown in Fig. 6. Raw TSD data (available in Appendix S1) were first filtered in MATLAB to remove outliers according to the median method. Next, TSD values exceeding 0.15 were excluded as outliers from the time-course data in Fig. 6. Cultures grown in light/dark cycles (both autotrophic and mixotrophic) exhibited bimodal deviations from the initial phenotypes and including the time-course outliers still produced the bimodal shape. The highest values of TSD occurred at 6 and 18 h, with a near return to the initial phenotypes at 12 h and a full return to the initial phenotypes at 24 h. Fourth-order polynomials were then fitted through the time-course data for each growth condition in Fig. 6. These high-order polynomials were chosen because of their ability to detect a bimodal deviation, should it exist. As shown in Fig. 6CD, the bimodal deviation did not occur for the autotrophic and heterotrophic cultures grown in the dark and the final phenotypes (after 24 h) did not return to the initial phenotypes.

### Correlation of individual Raman bands with metabolites and their levels analyzed by well-established analytical approaches

The nitrogen-limited cultures (0–100% of BG-11 medium nitrate; continuous light [20 µE]), described in earlier chemometric studies, were used in further phenotype validation experiments using analytical methods described in the Materials and Methods section. The purpose was to correlate individual Raman band intensities with specific biomolecules and results from other, widely accepted analytical methods (e.g., GC-FID, UPLC, spectroscopy).

#### Glycogen

Previous studies reported an activation of glycogen synthesis during nitrogen-limiting conditions (Osanai et al., 2006; Osanai et al., 2014; Yoo et al., 2007; Hasunuma et al., 2013; Adebiyi, Jazmin & Young, 2015). Therefore, glycogen levels were studied by comparing glycogen assigned bands in Raman spectroscopy (Movasaghi, Rehman & Rehman, 2007; Kamemoto et al., 2010) and an established spectrophotometric method (Osanai et al., 2011). As expected, glycogen levels increased in response to nitrogen deprivation by using both methods (Fig. S6). Cultures given 0.88 mM nitrate (5% of BG-11) showed about 2.5-fold increase in glycogen levels compared to cells grown at 17.6 mM (100% of BG-11) nitrate. Cultures grown in nitrate concentrations of 1.76 mM (10%), 13.2 mM (75%) and 16.72 mM (95%) showed increased glycogen levels by about 1.5-fold. However, growth in 3.52 mM (20%) and 8.8 mM (50%) nitrate showed no additional accumulation of glycogen.

Raman band assignments for glycogen (Movasaghi, Rehman & Rehman, 2007; Kamemoto et al., 2010) were correlated with the spectrophotometric measurements (Fig. S6). Correlation was observed with the glycogen-related bands at 1150 cm^−1^ (*R* = 0.82) and at 1,155 cm^−1^ (*R* = 0.80), suggesting that Raman band analysis may be used for determining glycogen levels. Although Raman spectroscopy cannot be used to obtain absolute concentrations of molecules, the trends reflecting changes in glycogen levels in response to varying nitrate availability were comparable between the two methods.

#### Amino acids

Proteins and amino acids represent the major nitrogen-containing compounds in a cell. Previous studies have demonstrated that protein and amino acid levels decrease dramatically with prolonged nitrate limitation (Osanai et al., 2006; Osanai et al., 2014; Hasunuma et al., 2013; Hauf et al., 2013; Adebiyi, Jazmin & Young, 2015). The levels of total amino acids (protein-derived and free amino acids) were determined by both Raman spectroscopy (De Gelder et al., 2007; Zhu et al., 2011) and UPLC coupled with fluorescence detection (Fig. S7). Individual Raman bands used for each amino acid are given in Table S1 in Appendix S2. Correlations that varied between *R* = 0.6 − 0.91 were observed between Raman spectroscopy and UPLC data (Fig. S8) for the majority of amino acids. Only Gly and Phe had moderate correlations, where the correlation coefficient varied from *R* = 0.44 − 0.54. It is important to note that some amino acids such as Cys and Trp are degraded during acidic hydrolysis and are not detected by UPLC (Olcott & Fraenkel-Conrat, 1947; Hugli & Moore, 1972; Yamada, Moriya & Tsugita, 1991). Raman band data enabled the prediction of expression trend of these two amino acids (Fig. S9), suggesting also a decrease in Cys and Trp levels during prolonged nitrate starvation. In addition, Asn and Gln are converted to Asp and Glu during acidic protein hydrolysis and, as such, Asp and Glu levels reflect Asp/Asn and Glu/Gln levels, respectively (Holt, Milligan & Roxburgh, 1971; Hesse & Weller, 2016). Our Raman spectroscopy and UPLC measurements are in good agreement, and the results are consistent with previously published metabolic phenotypes reflecting metabolic adaptations to nitrogen deprivation (Hauf et al., 2013; Osanai et al., 2014).

#### Fatty acids

Nitrogen starvation leads to oxidative stress, which causes damage to membrane lipids (Allen & Smith, 1969; Stevens, Balkwill & Paone, 1981; Wanner et al., 1986); (Kumar Saha, Uma & Subramanian, 2003). The levels of total saturated and unsaturated fatty acids were determined by both GC-FID (Fig. S10A) and Raman spectroscopy. Overall, decreases in fatty acid levels were observed at low nitrate concentrations (0.88 mM and 1.76 mM; 5% and 10% of BG-11 medium, respectively). Moderate correlations were obtained between Raman assigned bands to fatty acids (Movasaghi, Rehman & Rehman, 2007) and GC-FID data. While the correlation coefficients were somewhat low (R ∼0.5), this may be due to GC-FID-measured fatty acids at 13.2 mM (75%) nitrate that do not appear to fit with the time-course data, suggesting a problem with this particular GC-FID data point (Fig. S10). For example, with measurements at 13.2 mM nitrate included in the dataset, the correlation with the Raman band intensity at 1,078 cm^−1^ (I_1078_) was *R* < 0.5. With the data at 13.2 mM nitrate removed, the correlation improved, *R* > 0.85.

#### Chlorophyll a

Nitrogen deprivation stress leads to damage of photosynthetic components, including chlorophyll degradation, resulting in chlorosis (Krasikov et al., 2012; Hasunuma et al., 2013). Levels of photosynthetic pigments can be measured by both Raman spectroscopy and using spectrophotometric methods (Sinetova et al., 2012). As such, changes in chlorophyll levels were evaluated by these two approaches (Fig. S11 in Appendix S2). Raman signals corresponding to chlorophyll a (Wood et al., 2005; Jehlicka, Edwards & Oren, 2014) show a correlation with the spectrophotometric measurements (Fig. S11). The Raman band assignment 1,239 cm^−1^ had the highest correlation coefficient (*R* = 0.92) with the spectroscopic data. As expected, chlorophyll a levels decreased with the severity of nitrogen deprivation. Based on these results, Raman spectroscopy may also be used to quantify the levels of pigments in *Synechocystis*.

## Discussion

Raman spectroscopy with Rametrix™ was used as an analysis tool to study metabolic changes related to different growth conditions in *Synechocystis sp.* PCC6803. Two methods were explored here: (i) broad phenotype changes to external stimuli using chemometric approaches and (ii) the correlation of individual Raman bands with specific biomolecules, with validation by accepted analytical methods. Raman spectroscopy is rapid, non-destructive, and reliable for the characterization of changes in metabolic phenotypes as a response to changes in growth conditions. Clear distinctions were observed between cells grown in the absence or presence of glucose and in various light conditions. In most cases, Raman spectral data were found to represent changes in the biomolecular composition of cells well and enabled the differentiation of phenotypes. For example, clear statistically relevant differences were found between phenotypes of autotrophic cultures growing in BG-11 medium and mixotrophic/heterotrophic cultures growing in BG-11 medium with 5 mM glucose. Given an unknown Raman spectrum of *Synechocystis* growing in one of these environments, Rametrix™ PRO could determine that environment with 95.3% accuracy (sensitivity = 93.4%; specificity = 96.9%). The observation of glucose-induced phenotypes agrees with previous studies (Williams, 1988; Vermaas, 1996; Yoo et al., 2007; Lopo et al., 2012; Yu et al., 2013). It was reported previously that glucose has an effect on the *Synechocystis* growth rate as it activates other sets of metabolic pathways (Williams, 1988; Yoo et al., 2007; Lopo et al., 2012; Yu et al., 2013), which should lead to phenotype changes detectable by Raman spectroscopy.

Phenotype changes due to different illumination were also found through Rametrix™ LITE clustering of Raman spectral data, confirmed for the continuous light phenotypes through statistical analyses (ANOVA and pairwise comparisons), and predictable over random chance using Rametrix™ PRO (including 100% sensitivity and specificity for the continuous light phenotype). Previous studies have revealed that light is one of the most important environmental factors relevant to growth of photosynthetic organisms (Mohamed & Jansson, 1989; Anderson & McIntosh, 1991; Vermaas, 1996; Lopo et al., 2012; Dechatiwongse et al., 2014) . Light plays fundamental roles including, but not limited to, altering gene expression (Mohamed & Jansson, 1989; Gill et al., 2002), initiating phototaxis (Mullineaux, 2001), and resetting circadian rhythm (Mullineaux, 2001; Kucho et al., 2005). In addition, excess of light (Aro, Virgin & Andersson, 1993; Zer & Ohad, 1995; Vermaas, 1996; Allakhverdiev & Murata, 2004) and UV light (Vass, Kirilovsky & Etienne, 1999) might induce damage to the photosystem II. These light excess phenotypic changes appear to be the most easily detected by Raman spectroscopy and Rametrix™.

While clear cluster separations were not observed with Rametrix™ LITE models for nitrogen-limiting phenotypes, ANOVA and pairwise comparisons confirmed phenotype changes. In addition, Rametrix™ PRO was able to predict the level of nitrate in the culture medium with accuracy, sensitivity, and specificity greater than by random chance. Furthermore, analyses by accepted analytical methods (e.g., GC-FID, UPLC, spectroscopy, etc.) confirmed changes in glycogen, fatty acids, amino acids, and chlorophyll a, which were then correlated with specific Raman bands in good agreement. These results agree with several literature reports suggesting nitrogen-limitation induced phenotype changes. In particular, *Synechocystis* cells respond to nitrate starvation by reprogramming central carbon and nitrogen metabolism, which is reflected by changes in the levels of various high abundance macromolecules and metabolites, including glycogen (Osanai et al., 2006; Yoo et al., 2007; Hasunuma et al., 2013; Adebiyi, Jazmin & Young, 2015), proteins, amino acids (Osanai et al., 2006; Hasunuma et al., 2013; Hauf et al., 2013; Adebiyi, Jazmin & Young, 2015), lipids (Allen & Smith, 1969; Stevens, Balkwill & Paone, 1981; Wanner et al., 1986; Kumar Saha, Uma & Subramanian, 2003), and photosynthetic pigments (Holt, Milligan & Roxburgh, 1971). All of these changes and phenotypes were confirmed here as the metabolites and macromolecules could be reliably detected from Raman spectra. Previous studies also revealed that nitrogen limitation induces specific cellular responses, including nitrogen assimilation up-regulation as well as degradation of chlorophyll, phycobilisomes, and light harvesting complexes, leading to chlorosis (Grossman et al., 1994; Krasikov et al., 2012; Hasunuma et al., 2013), which is consistent with our results (Fig. S5B in Appendix S2). In addition, cyanobacteria placed under low nitrogen stress conditions degrade intracellular proteins to amino acids to mobilize nitrogen and to catabolize amino acids to provide carbon precursors for sugar and glycogen synthesis (Osanai et al., 2006; Osanai et al., 2014; Hasunuma et al., 2013; Adebiyi, Jazmin & Young, 2015).

Regarding the circadian rhythm results, our observations confirmed our hypothesis that TSD values (representative of phenotypes) would return to their initial values after 24 h for cultures grown in light/dark (12 h/12 h) cycles. This was true of both autotrophic and heterotrophic cultures. The bimodal nature of this 24-hour cycle was unexpected and warrants further investigations. We also noted more extreme variances for light/dark grown cultures compared to cultures grown in the dark. This could be indicative of a higher degree of heterogeneity for light/dark grown cultures, based on whether analyzed cells were closer to the flask exterior or center when sampled. This has been known in the literature to play a role in metabolic activity (Kumar et al., 2011).

Overall, correlation was observed (*R* > 0.7) between Raman band intensities and the data obtained by well-established analytical methods. As such, Raman spectroscopy is suitable for comparative studies when relative levels of metabolites and macromolecules need to be determined. However, Raman spectra are highly convoluted and finding correlating bands may not always be possible. In addition, preliminary experiments such as the ones described in this study, should be performed for new cell types and culturing environments. Specific bands may become hidden due to potentially interfering bands belonging to other biomolecules. The presence of interfering compounds will be highly dependent on the type of the sample. Once the correlating bands are established, Raman spectroscopy can be used for nearly any type of high-throughput screening of metabolic phenotypes.

## Conclusions

Raman spectroscopy with the Rametrix™ Toolboxes for MATLAB® and statistical tests showed the ability to distinguish among *Synechocystis* PCC6803 phenotypes derived from growth (i) in the presence/absence of glucose, (ii) under different illumination, (iii) under nitrogen deprivation, and (iv) over a circadian rhythm cycle. In particular, given a Raman spectrum of *Synechocystis* cells grown in unknown culture conditions, the presence/absence of glucose in the growth medium was determined with 95% accuracy (93% sensitivity and 97% specificity). Whether the culture was grown in the dark, light/dark, or continuous light was determined with 89.5%, 78%, and 100% accuracies, respectively. The ability to detect nitrogen limitation conditions was better than random chance, and analysis of circadian rhythms over 24 h light/dark cycles showed phenotypes that deviated and returned to their initial states. In addition, several macromolecules and amino acids were correlated with specific Raman bands. We anticipate Raman spectroscopy and Rametrix™ will enable rapid and cost-efficient phenotyping of *Synechocystis* cultures, and that the techniques described throughout may be applied to other organisms.

##  Supplemental Information

10.7717/peerj.8585/supp-1 Appendix S1 Raman band assignments; Rametrix™ and statistical calculations

10.7717/peerj.8585/supp-2 Appendix S2 Results of analytical experiments

10.7717/peerj.8585/supp-3 Data S1 Raman scan files and analytical experiment measurements

## Additional Information and Declarations

### Data Availability

The following information was supplied regarding data availability: All Raman spectra and raw data measurements from analytical experiments are available as a Supplemental File. The RametrixTM LITE Toolbox is available through GitHub (https://github.com/SengerLab/RametrixLITEToolbox) and the RametrixTM PRO Toolbox is also shared through GitHub (https://github.com/SengerLab/RametrixPROToolbox). Both are shared under the MIT license.
