# Supplementary material for: Characterizing glucose, illumination, and nitrogen-deprivation phenotypes of Synechocystis PCC6803 with Raman spectroscopy
Source: PeerJ. 2020 Mar 30;8:e8585. doi: 10.7717/peerj.8585 (PMC7115749; doi:10.7717/peerj.8585)
Supplement: Appendix S2 [file peerj-08-8585-s002.pdf]

Supplementary Appendix 2

Characterizing metabolic stress-induced phenotypes of *Synechocystis* PCC6803 with Raman spectroscopy

Imen Tanniche[1], Eva Collakova[2], Cynthia Denbow[2], Ryan S. Senger*[1,3]

[1] Department of Biological Systems Engineering; Virginia Tech; Blacksburg, VA

[2] School of Plant & Environmental Sciences; Virginia Tech; Blacksburg, VA

[3] Department of Chemical Engineering; Virginia Tech; Blacksburg, VA

* Corresponding Author
1230 Washington St.
301C HABB1
Blacksburg, VA 24061
Email: senger@vt.edu
Phone: 540-231-9501

Note: Figures **S1-S4** are contained in Supplementary Appendix 1

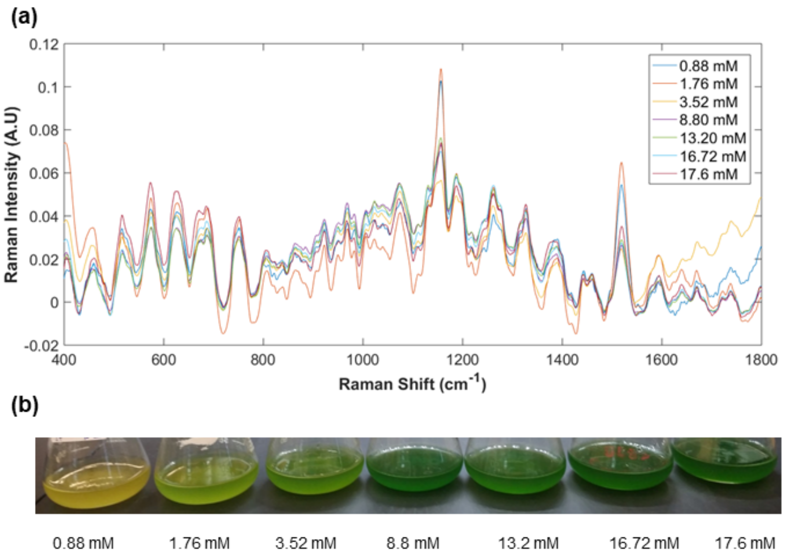

Figure S5. *Synechocystis sp*. PCC6803 culture growth under different concentrations of nitrate. (a) Averaged spectra baselined and vector normalized over the range 400-1800 cm^{-1}. (b) Visual comparison of bleaching of cells growing under low concentrations of nitrate, where 17.6 mM is nitrate concentration in unaltered BG-11 medium.

Conversion to percentages: 5% = 0.88 mM; 10% = 1.76 mM; 20% = 3.52 mM; 50% = 8.8 mM; 75% = 13.2 mM; 95% = 16.72 mM; 100% = 17.6 mM

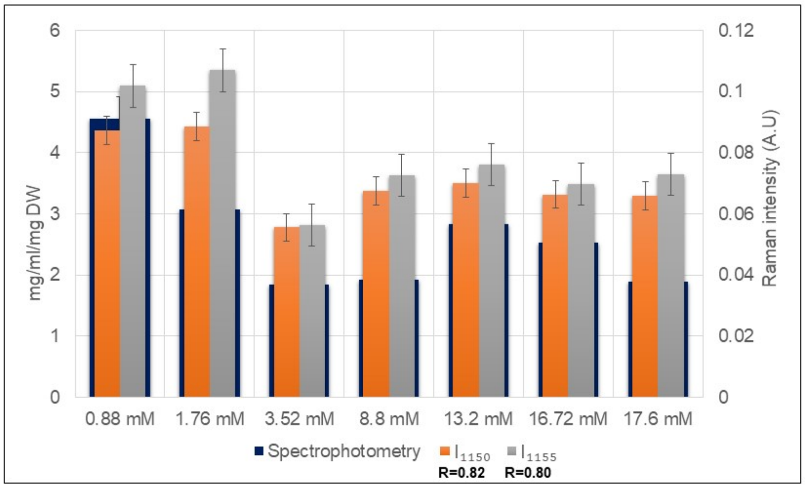

Figure S6. Glycogen analysis and Raman spectroscopy in in *Synechocystis sp*. PCC 6803 cells grown under different concentrations of nitrate. Correlation coefficients (R) between Raman bands and glycogen level are represented.

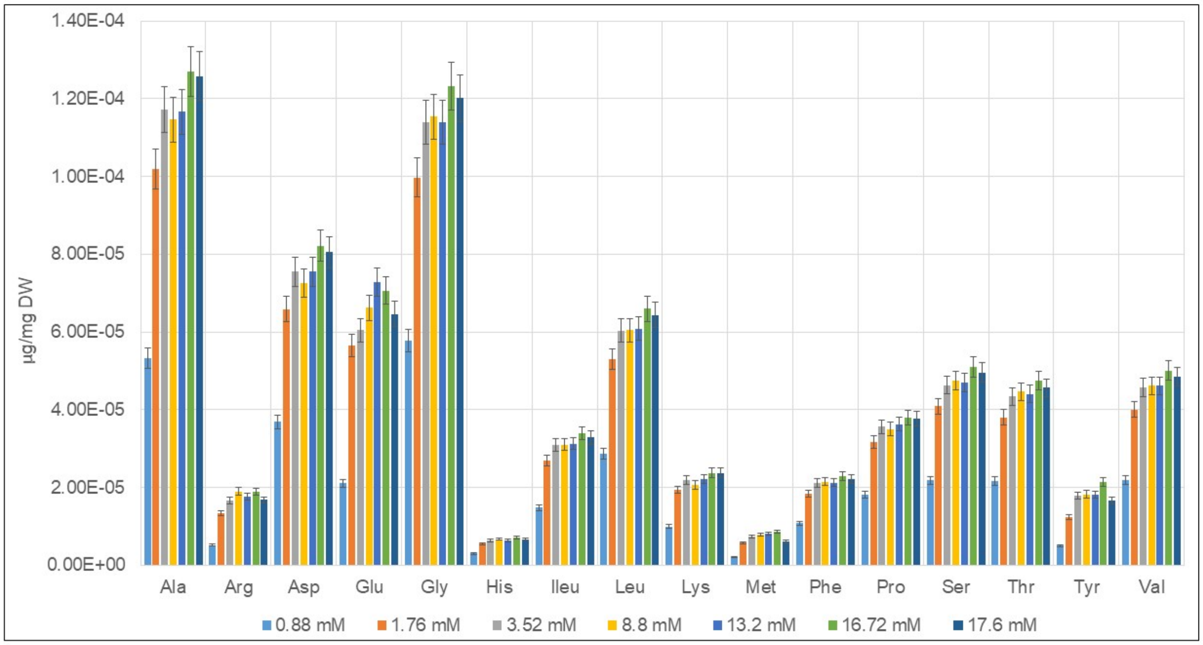

Figure S7. Levels of amino acids under nitrogen limitation conditions. Data represent means ± standard deviation (SD) of values from three independent experiments. *Synechocystis sp*. PCC6803 cells under 17.6 mM conditions represents the regular concentration of nitrate in BG-11 medium.

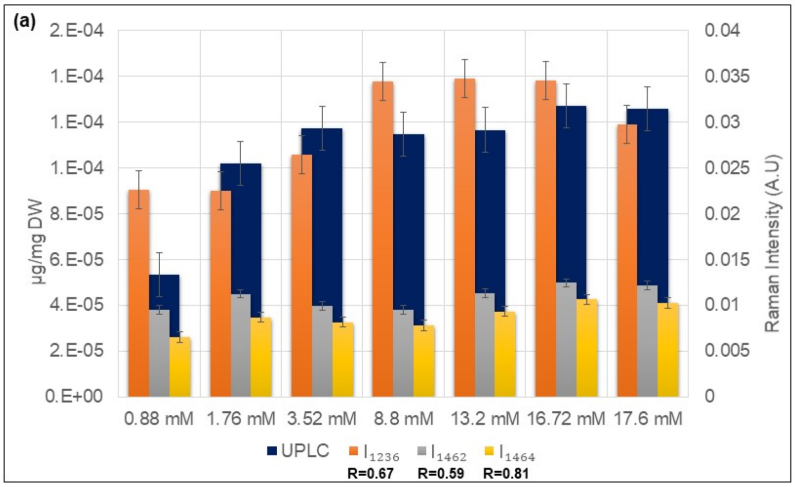

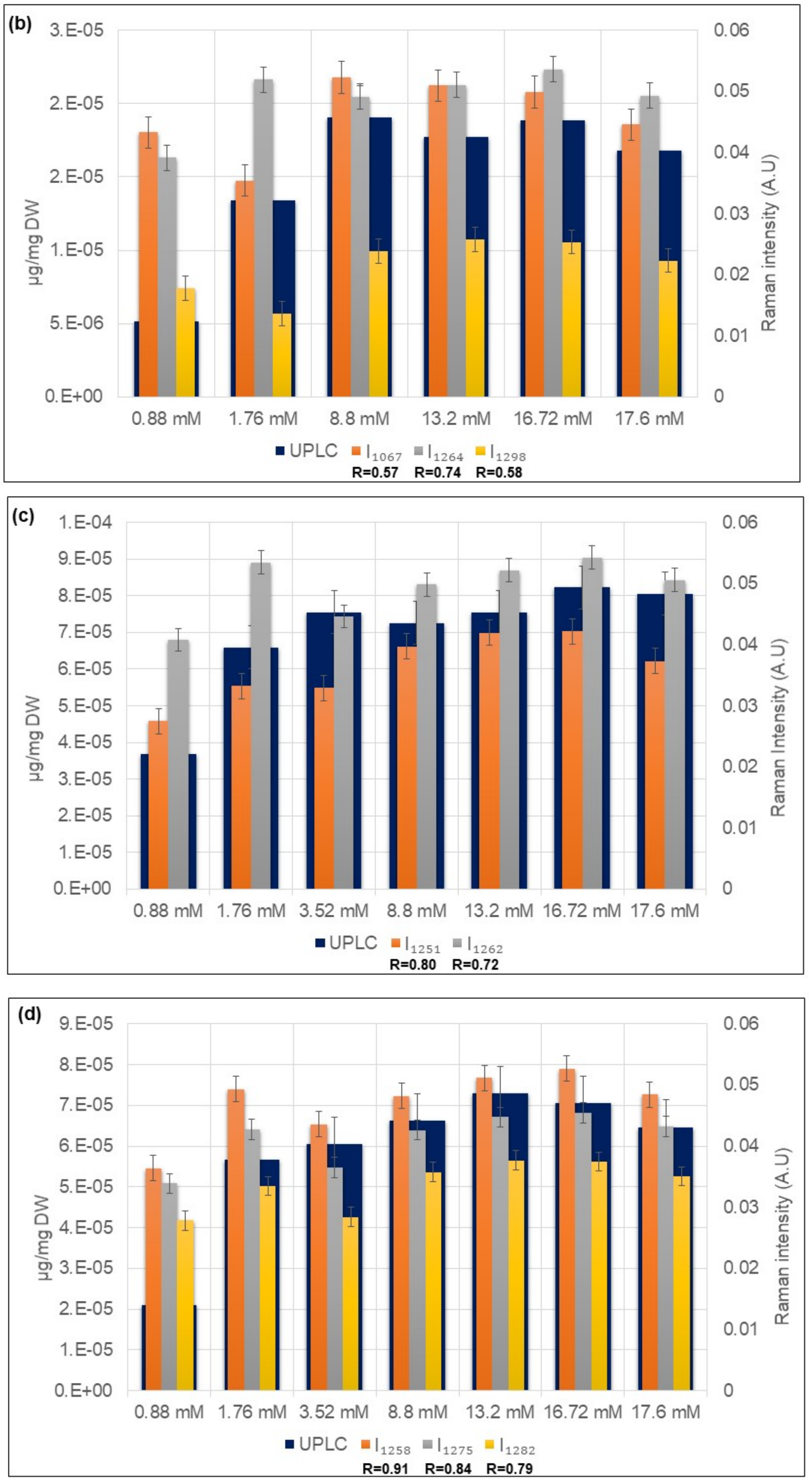
(b)
3.E-05
2.E-05
2.E-05
1.E-05
5.E-06
0.E+00
µg/mg DW
0.06
0.05
0.04
0.03
0.02
0.01
0
Raman intensity (A.U)
0.88 mM
1.76 mM
8.8 mM
13.2 mM
16.72 mM
17.6 mM
UPLC I_{1067} I_{1264} I_{1298}
R=0.57 R=0.74 R=0.58
(c)
1.E-04
9.E-05
8.E-05
7.E-05
6.E-05
5.E-05
4.E-05
3.E-05
2.E-05
1.E-05
0.E+00
µg/mg DW
0.06
0.05
0.04
0.03
0.02
0.01
0
Raman Intensity (A.U)
0.88 mM
1.76 mM
3.52 mM
8.8 mM
13.2 mM
16.72 mM
17.6 mM
UPLC I_{1251} I_{1262}
R=0.80 R=0.72
(d)
9.E-05
8.E-05
7.E-05
6.E-05
5.E-05
4.E-05
3.E-05
2.E-05
1.E-05
0.E+00
µg/mg DW
0.06
0.05
0.04
0.03
0.02
0.01
0
Raman intensity (A.U)
0.88 mM
1.76 mM
3.52 mM
8.8 mM
13.2 mM
16.72 mM
17.6 mM
UPLC I_{1258} I_{1275} I_{1282}
R=0.91 R=0.84 R=0.79

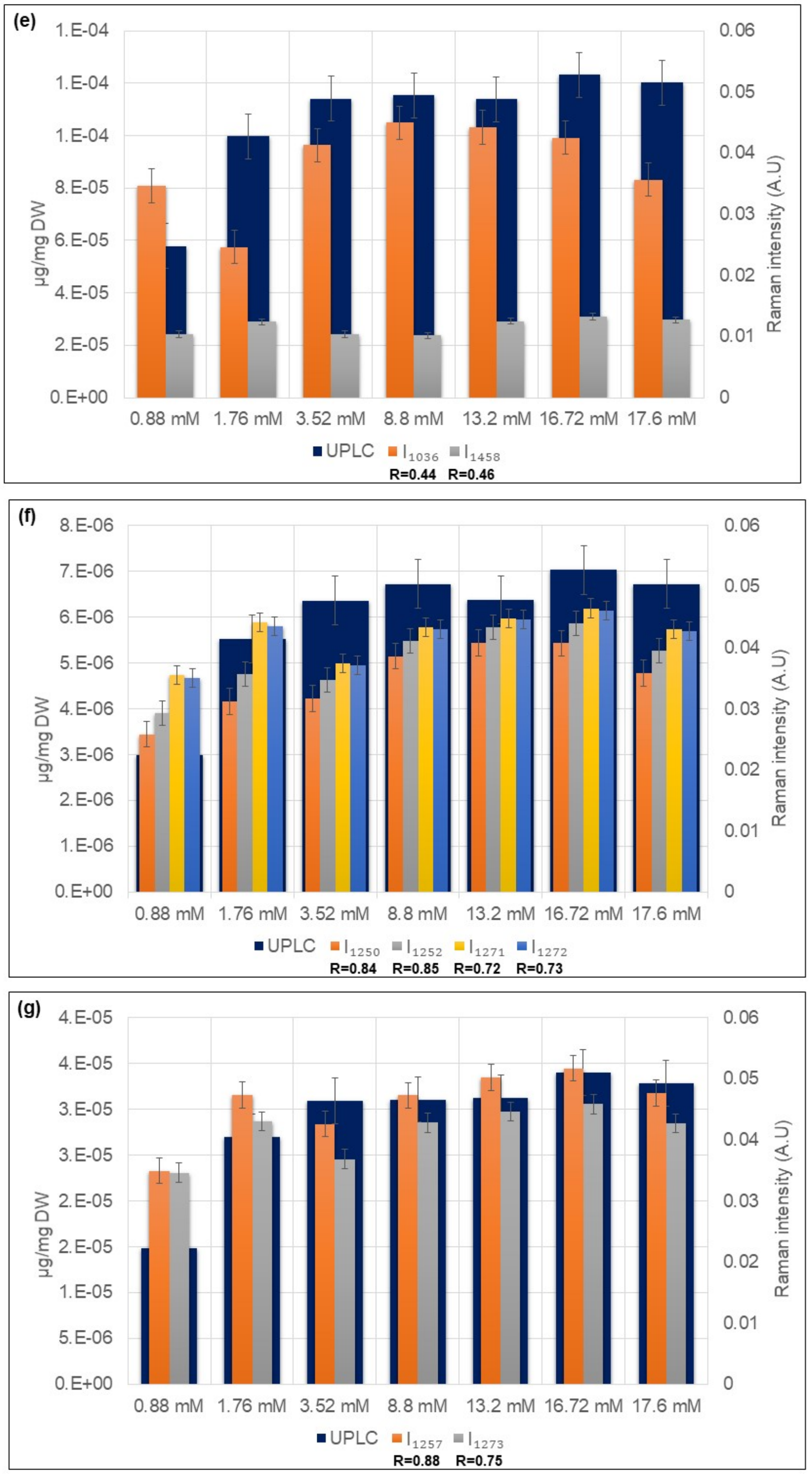
(e)
µg/mg DW
Raman intensity (A.U)
1.E-04
8.E-05
6.E-05
4.E-05
2.E-05
0.E+00
0.06
0.05
0.04
0.03
0.02
0.01
0
0.88 mM
1.76 mM
3.52 mM
8.8 mM
13.2 mM
16.72 mM
17.6 mM
UPLC
I_{1036}
I_{1458}
R=0.44
R=0.46
(f)
µg/mg DW
Raman intensity (A.U)
8.E-06
7.E-06
6.E-06
5.E-06
4.E-06
3.E-06
2.E-06
1.E-06
0.E+00
0.06
0.05
0.04
0.03
0.02
0.01
0
0.88 mM
1.76 mM
3.52 mM
8.8 mM
13.2 mM
16.72 mM
17.6 mM
UPLC
I_{1250}
I_{1252}
I_{1271}
I_{1272}
R=0.84
R=0.85
R=0.72
R=0.73
(g)
µg/mg DW
Raman intensity (A.U)
4.E-05
3.E-05
2.E-05
1.E-05
5.E-06
0.E+00
0.06
0.05
0.04
0.03
0.02
0.01
0
0.88 mM
1.76 mM
3.52 mM
8.8 mM
13.2 mM
16.72 mM
17.6 mM
UPLC
I_{1257}
I_{1273}
R=0.88
R=0.75

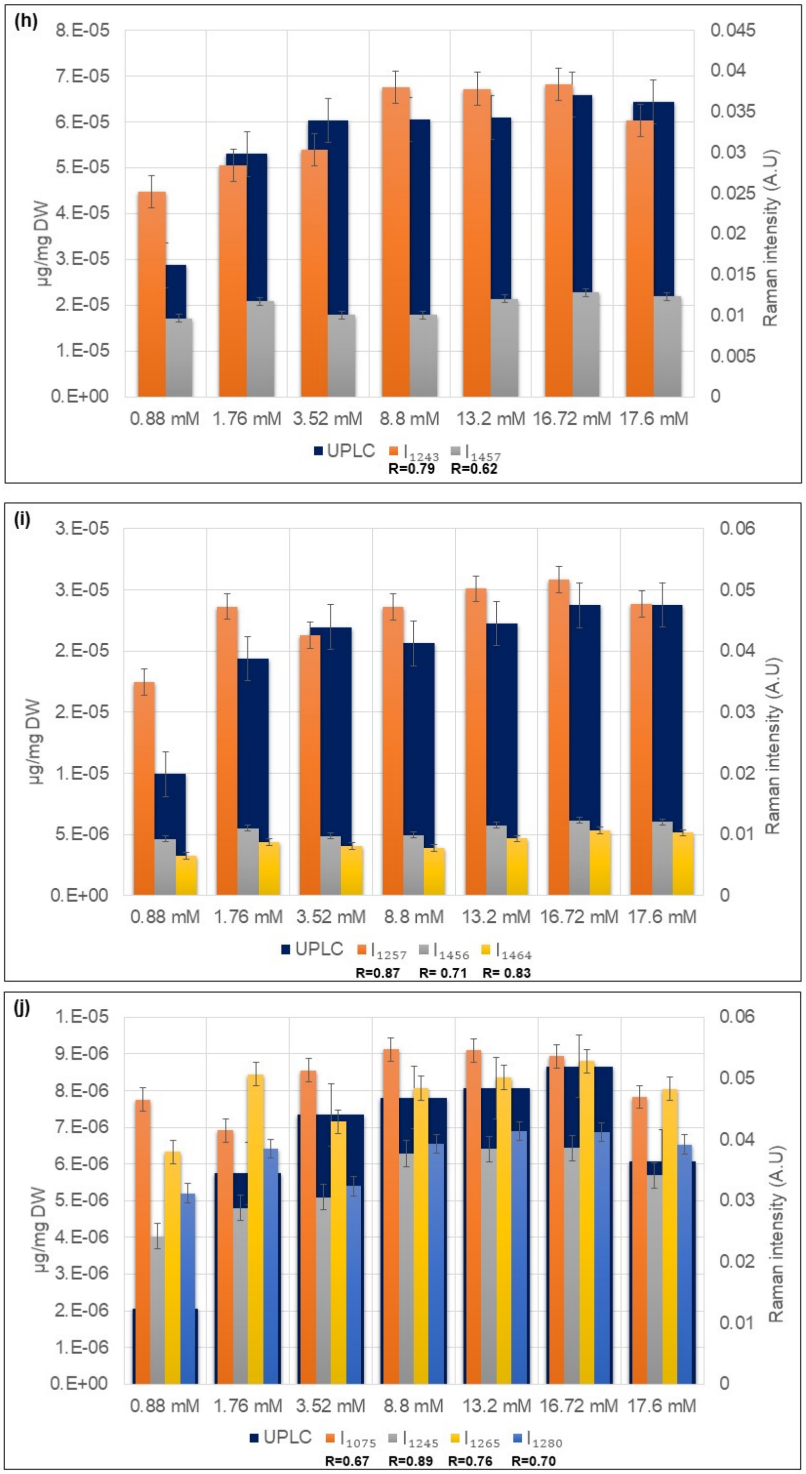
(h)
µg/mg DW
Raman intensity (A.U)
0.88 mM 1.76 mM 3.52 mM 8.8 mM 13.2 mM 16.72 mM 17.6 mM
UPLC
I_{1243} R=0.79
I_{1457} R=0.62
(i)
µg/mg DW
Raman intensity (A.U)
0.88 mM 1.76 mM 3.52 mM 8.8 mM 13.2 mM 16.72 mM 17.6 mM
UPLC
I_{1257} R=0.87
I_{1456} R= 0.71
I_{1464} R= 0.83
(j)
µg/mg DW
Raman intensity (A.U)
0.88 mM 1.76 mM 3.52 mM 8.8 mM 13.2 mM 16.72 mM 17.6 mM
UPLC
I_{1075} R=0.67
I_{1245} R=0.89
I_{1265} R=0.76
I_{1280} R=0.70

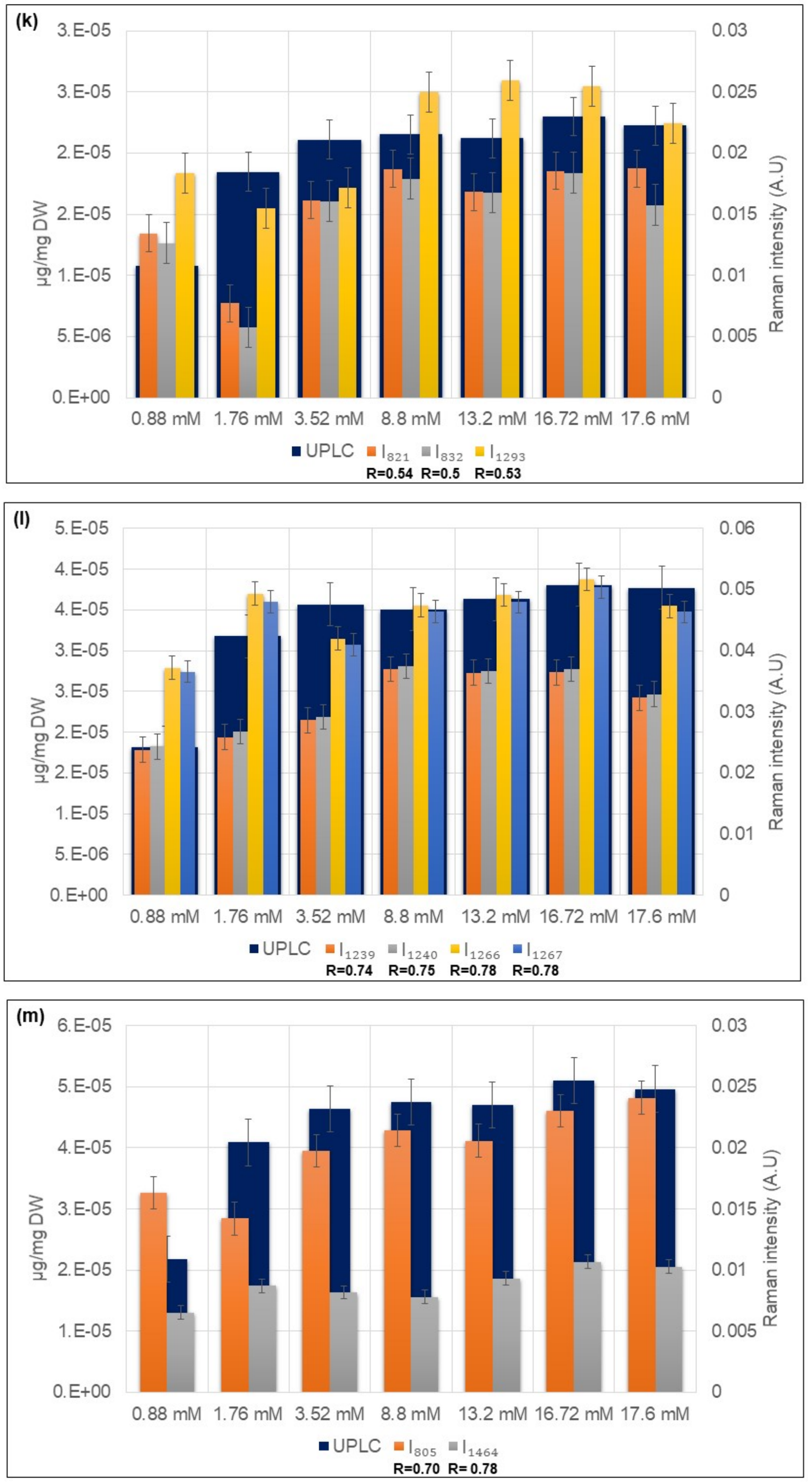
(k)
3.E-05
3.E-05
2.E-05
2.E-05
1.E-05
5.E-06
0.E+00
µg/mg DW
0.03
0.025
0.02
0.015
0.01
0.005
0
Raman intensity (A.U)
0.88 mM 1.76 mM 3.52 mM 8.8 mM 13.2 mM 16.72 mM 17.6 mM
UPLC I_{821} I_{832} I_{1293}
R=0.54 R=0.5 R=0.53
(l)
5.E-05
4.E-05
4.E-05
3.E-05
3.E-05
2.E-05
2.E-05
1.E-05
5.E-06
0.E+00
µg/mg DW
0.06
0.05
0.04
0.03
0.02
0.01
0
Raman intensity (A.U)
0.88 mM 1.76 mM 3.52 mM 8.8 mM 13.2 mM 16.72 mM 17.6 mM
UPLC I_{1239} I_{1240} I_{1266} I_{1267}
R=0.74 R=0.75 R=0.78 R=0.78
(m)
6.E-05
5.E-05
4.E-05
3.E-05
2.E-05
1.E-05
0.E+00
µg/mg DW
0.03
0.025
0.02
0.015
0.01
0.005
0
Raman intensity (A.U)
0.88 mM 1.76 mM 3.52 mM 8.8 mM 13.2 mM 16.72 mM 17.6 mM
UPLC I_{805} I_{1464}
R=0.70 R= 0.78

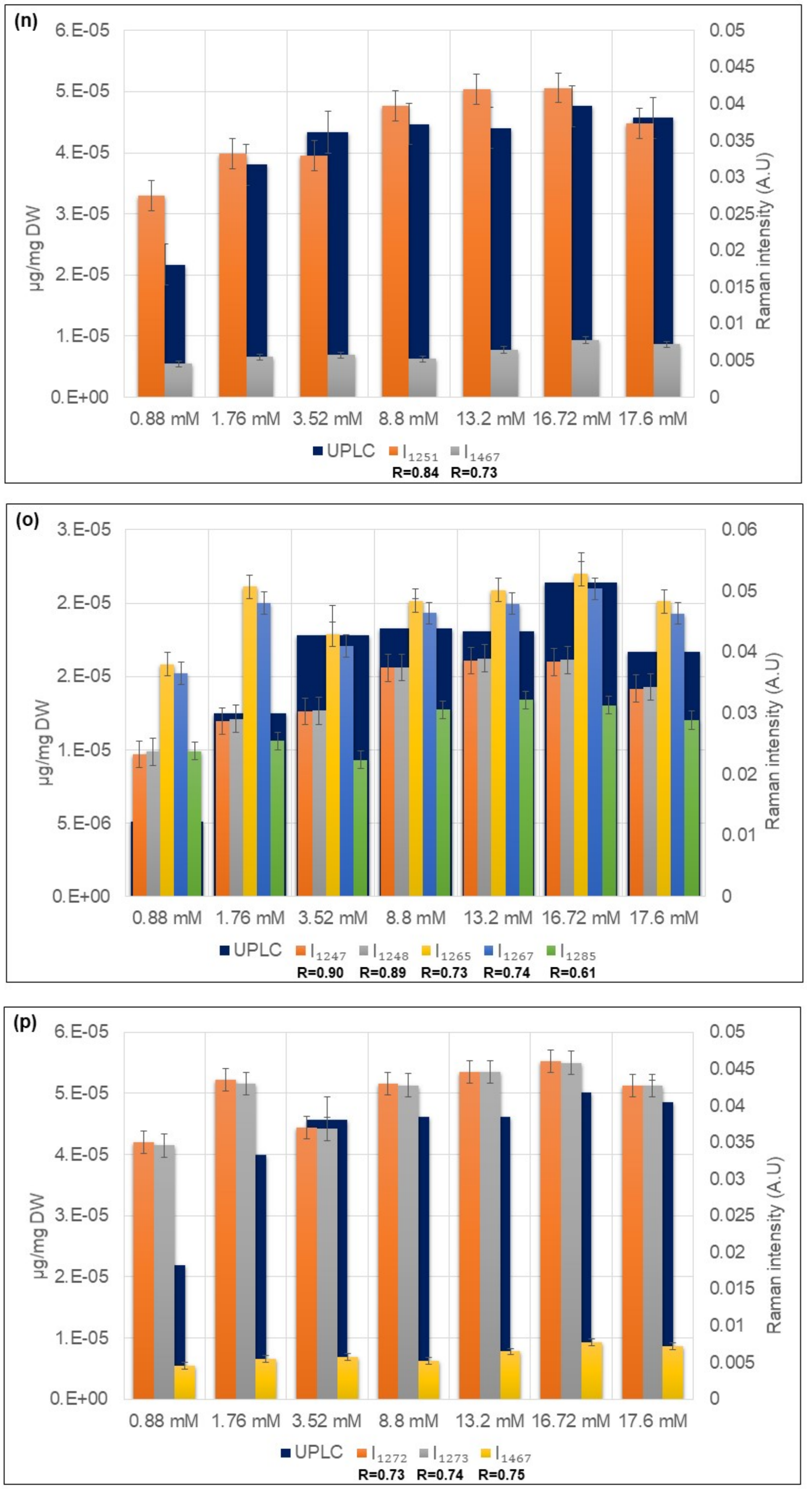

Figure S8. Correlation of Raman data with UPLC extraction of amino acids. (a) Alanine, (b) Arginine, (c) Aspartate/Asparagine, (d) Glutamate, (e) Glycine, (f) Histidine, (g) Isoleucine, (h) Leucine, (i) Lysine, (j) Methionine, (k) Phenylalanine, (l) Proline, (m) Serine, (n) Threonine, (o) Tyrosine, (p) Valine. Correlation coefficients (R) for each Raman band are represented.

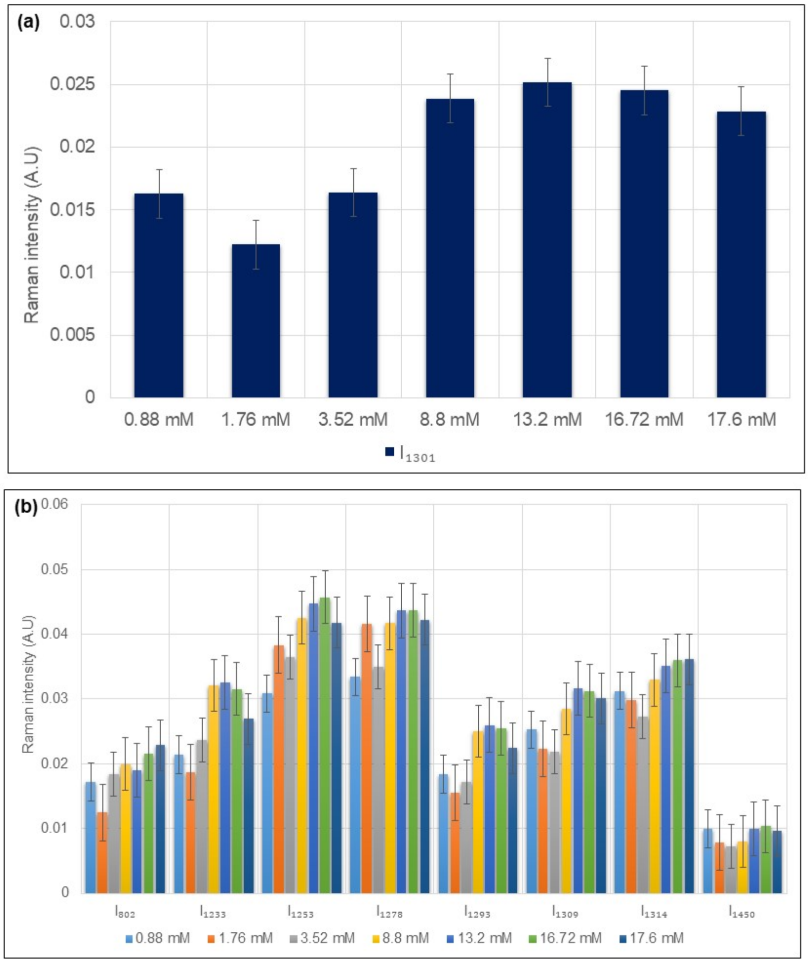

Figure S9. Raman bands predictions of amino acids non-resolved with UPLC. (a) Cysteine predictions, (b) Tryptophan predictions.

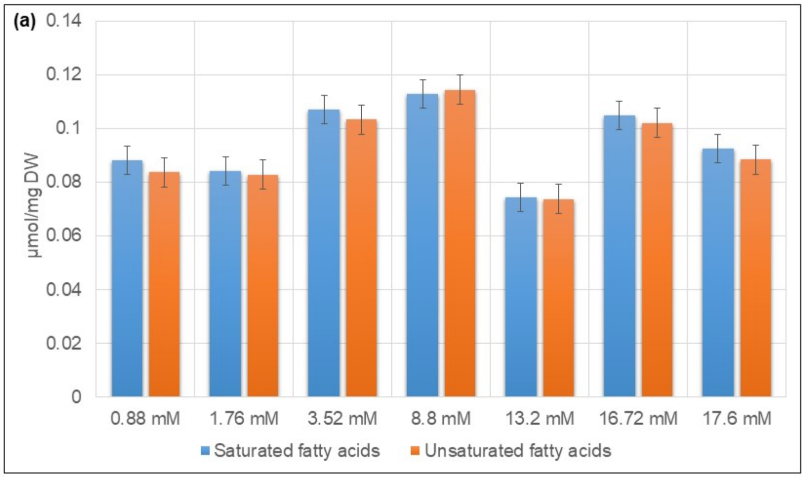

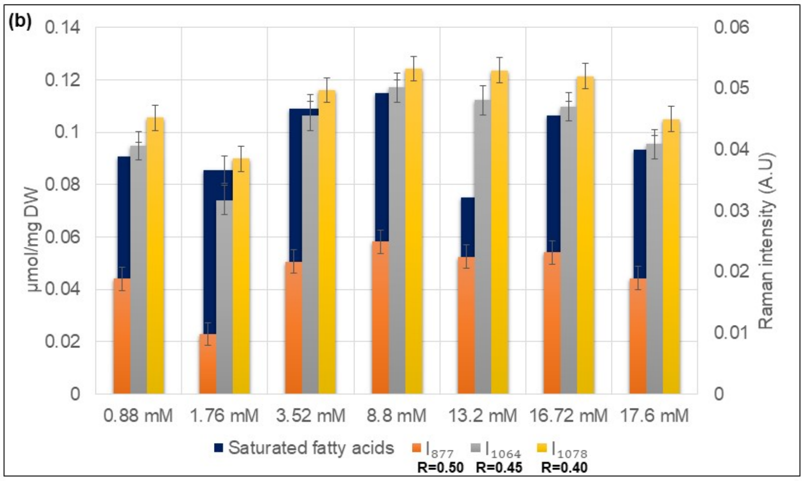

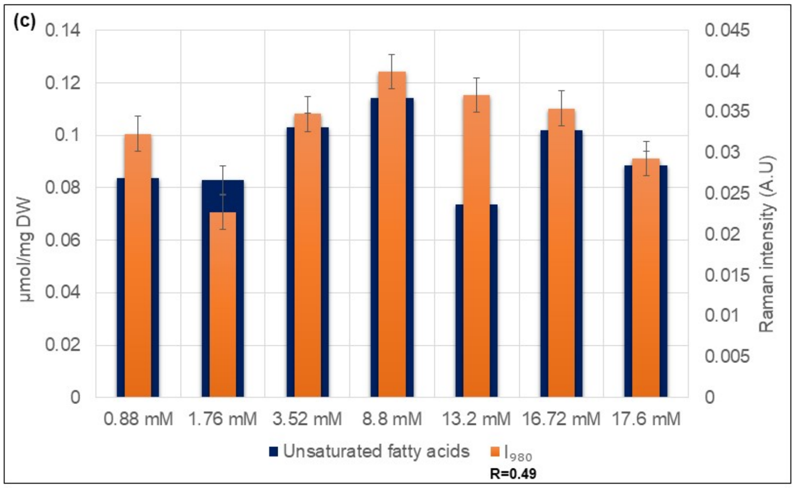

Figure S10. Fatty acid levels analysis with GC-FID and Raman spectroscopy in in *Synechocystis sp*. PCC 6803 cells grown under different concentrations of nitrate. (a) Total unsaturated fatty acid and total saturated fatty acids levels, (b) Correlation between Raman spectroscopy and GC-FID data of saturated fatty acids, (c) Correlation between Raman spectroscopy and GC-FID data of unsaturated fatty acids analysis. Correlation coefficients (R) for each Raman band are represented.

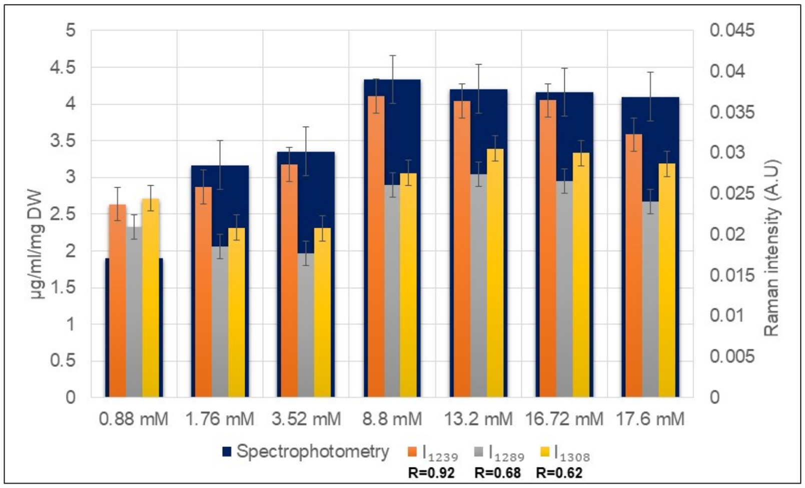

Figure S11. Chlorophyll a level and Raman spectroscopy in in *Synechocystis sp*. PCC 6803 cells grown under different concentrations of nitrate. Correlation coefficients (R) between Raman bands and chlorophyll a levels are represented.

Table S1. All Raman bands cited and tested for amino acids, chlorophyll a, glycogen and fatty acids.

**Biomolecules**	**Raman bands (cm^{-1}) and Correlation Coefficient (R)**	**References**
Ala	1236 (R = 0.67), 1462 (R = 0.59), 1464 (R = 0.81)	(1,2)
Arg	1067 (R = 0.57), 1264 (R = 0.74), 1298 (R = 0.58)	(1,2)
Asp/Asn	1251 (R = 0.80), 1262 (R = 0.72)	(2)
Cys*	1301	(2)
Glu/Gln	1258 (R = 0.91), 1275 (R = 0.84), 1282 (R = 0.79)	(1)
Gly	1036 (R = 0.44), 1458 (R = 0.46)	(2)
His	1250 (R = 0.84), 1252 (R = 0.85), 1271 (R = 0.72), 1272 (R = 0.73)	(1,2)
Ile	1257 (R = 0.88), 1273 (R = 0.75)	(2)
Leu	1243 (R = 0.79), 1457 (R = 0.62)	(2)
Lys	1257 (R = 0.87), 1456 (R = 0.71), 1464 (R = 0.83)	(2)
Met	1075 (R = 0.67), 1245 (R = 0.89), 1265 (R = 0.76), 1280 (R = 0.70)	(2)
Phe	821 (R = 0.54), 832 (R = 0.5), 1293 (R = 0.53)	(1,2)
Pro	1239 (R = 0.74), 1240 (R = 0.75), 1266 (R = 0.78), 1267 (R = 0.78)	(1,2)
Ser	805 (R = 0.70), 1464 (R = 0.78)	(2)
Thr	1251 (R = 0.84), 1467 (R = 0.73)	(2)

Trp*	802, 1233, 1253, 1278, 1293, 1309, 1314, 1450	(1,2)
Tyr	1247 (R = 0.90), 1248 (R = 0.89), 1265 (R = 0.73), 1267 (R = 0.74), 1285 (R = 0.61)	(1,2)
Val	1272 (R = 0.73), 1273 (R = 0.74), 1467 (R = 0.75)	(1,2)
Chlorophyll a	1239 (R = 0.92), 1289 (R = 0.68), 1308 (R = 0.62)	(3,4)
Glycogen	1150 (R = 0.82), 1155 (R = 0.80)	(5,6)
Unsaturated Fatty acids	980 (R = 0.49)	(6)
Saturated Fatty acids	877 (0.50), 1064 (0.45), 1078 (0.40)	(6)

* Values for Cys and Trp could not be obtained by the UPLC method used in this research. Only Raman predictions are provided.
** Possible overlapping bands between different biomolecules assigned Raman bands.

References

1. De Gelder J, De Gussem K, Vandenabeele P, Moens L. Reference database of Raman spectra of biological molecules. J Raman Spectrosc. 2007;38(9):1133–47.
2. Zhu G, Zhu X, Fan Q, Wan X. Raman spectra of amino acids and their aqueous solutions. Spectrochim Acta Mol Biomol Spectrosc. 2011;78(3):1187–95.
3. Jehlicka J, Edwards HG, Oren A. Raman spectroscopy of microbial pigments. Appl Env Microbiol. 2014;80(11):3286–95.
4. Wood BR, Heraud P, Stojkovic S, Morrison D, Beardall J, McNaughton D. A portable Raman acoustic levitation spectroscopic system for the identification and environmental monitoring of algal cells. Anal Chem. 2005;77(15):4955–61.
5. Kamemoto LE, Misra AK, Sharma SK, Goodman MT, Luk H, Dykes AC, et al. Near-infrared micro-Raman spectroscopy for in vitro detection of cervical cancer. Appl Spectrosc. 2010;64(3):255–61.
6. Movasaghi Z, Rehman S, Rehman IU. Raman Spectroscopy of Biological Tissues. Appl Spectrosc Rev. 2007;42(5):493–541.
