# Supplementary material for: Characterizing glucose, illumination, and nitrogen-deprivation phenotypes of Synechocystis PCC6803 with Raman spectroscopy
Source: PeerJ. 2020 Mar 30;8:e8585. doi: 10.7717/peerj.8585 (PMC7115749; doi:10.7717/peerj.8585)
Supplement: Data S1 [file peerj-08-8585-s003.zip › 00 - Readme.rtf]

Included here:1. Analytical experiments raw data.xlsx (this file contains raw data from GC-FID, UPLC, and spectroscopic measurement experiments for glycogen, fatty acids, amino acids, and chlorophyll a)2. CYB1 - CYB85 .SPC files (these are Raman spectroscopy raw data files)3. 00 - Fields File.csv (this file contains all information about Raman spectroscopy raw data files and is used with the RametrixTM LITE Toolbox v1.1)Instructions to read Raman Spectroscopy files:1. Download the RametrixTM LITE Toolbox v1.1 for MATLAB (https://github.com/SengerLab/RametrixLITEToolbox)2. Follow instructions there on how to load the fields file and Raman scan files.  Note: the v1.1 (and later versions) must be used.  These files will not work with v1.0.
